# 114. Application of a Machine Learning Algorithm to Routine Admission Laboratory Testing for Risk Adjustment among Patients with Suspected Severe Infection across 296 US Hospitals

**DOI:** 10.1093/ofid/ofaf695.046

**Published:** 2026-01-11

**Authors:** Daniel Rizk, Natalia Blanco, Katherine E Goodman, Larry Magder, Jonathan Baghdadi, Anthony Harris

**Affiliations:** University of Maryland, Baltimore, Maryland; University of Maryland Dept of Epidemiology and Public Health, Baltimore, MD; University of Maryland School of Medicine, Baltimore, Maryland; University of Maryland School of Medicine, Baltimore, Maryland; University of Maryland School of Medicine, Baltimore, Maryland; University of Maryland School of Medicine, Baltimore, Maryland

## Abstract

**Background:**

Severity of illness at the time of presentation is an important confounding variable in infectious disease research. This study validates and further develops an existing severity-of-illness model in patients with severe infection across 296 US hospitals. A prediction model at a single hospital was previously developed to estimate risk of in-hospital mortality using 9 routine admission lab tests from the University of Maryland Medical Center (PMID:31432440). We aimed to validate this modeling approach in a new patient population, and evaluate an alternative machine-learning implementation, across a national cohort.

**Figure:**
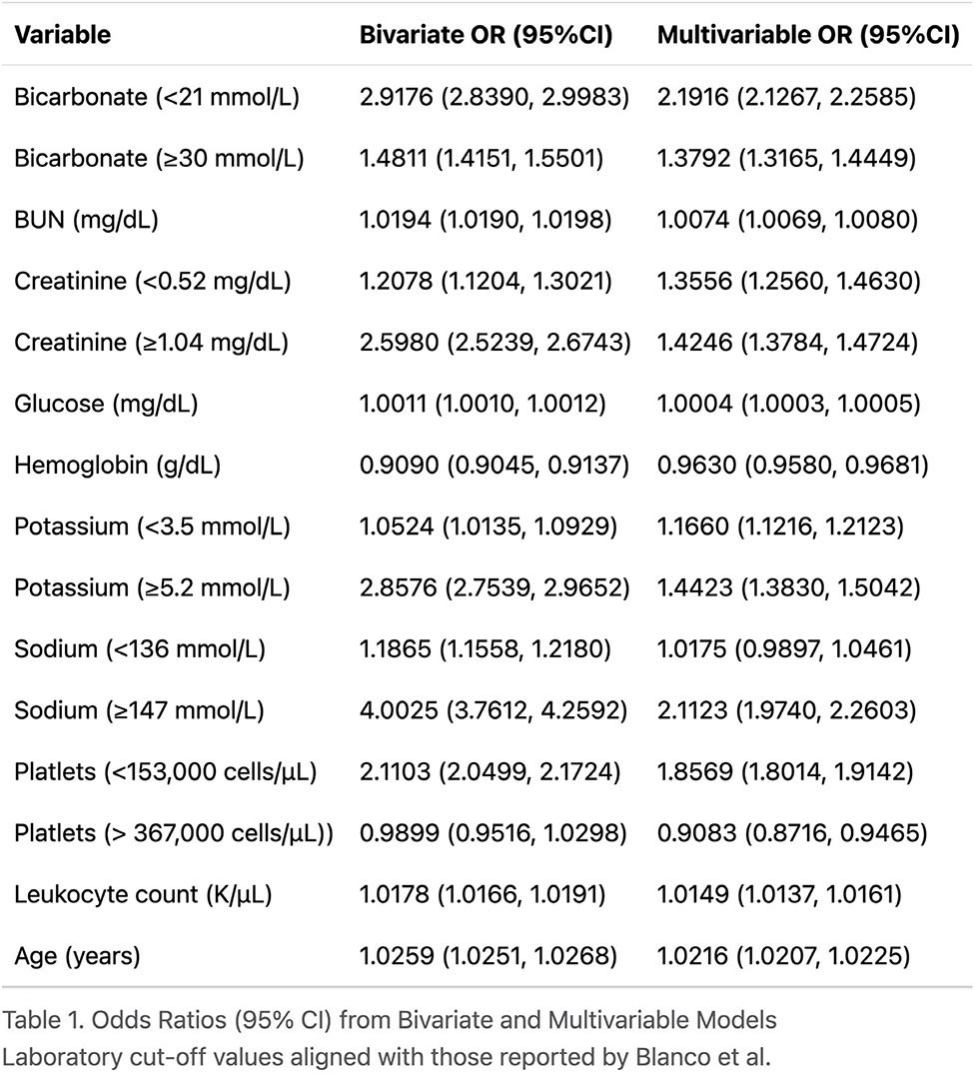
Odds Ratios (95%) CI from Bivariate and Multivariable Models

**Figure:**
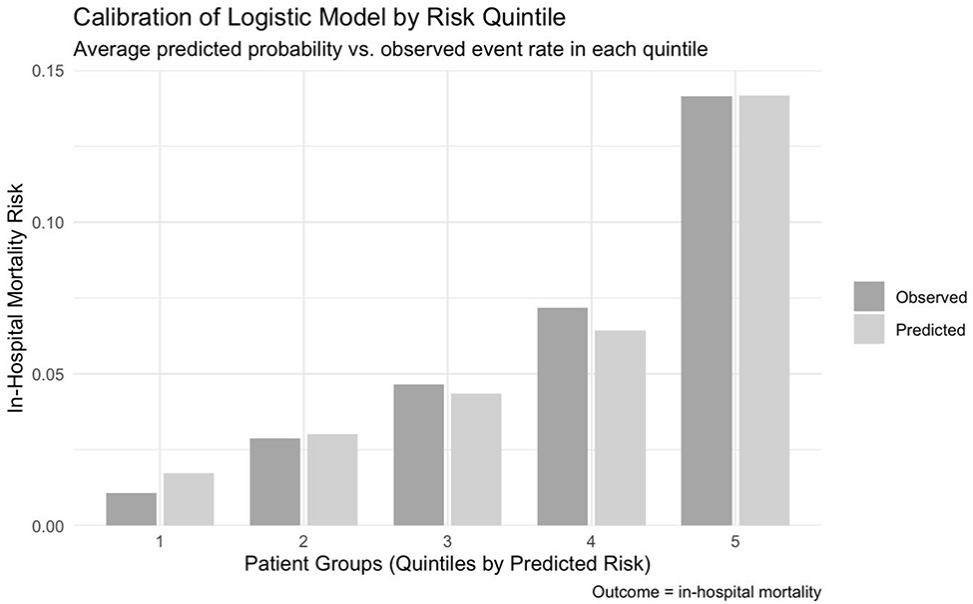
Laboratory cut-off values aligned with those reported by Blanco et al. Calibration of Logistic Model by Risk Quintile Average predicted probability vs. observed event rate in each quintile

**Methods:**

This retrospective cohort study included adults with suspected severe infection who were admitted to hospitals in the Premier Healthcare Database (2019–2023). Suspected severe infection was defined as the collection of blood cultures and administration of IV antibiotics according to the Sepsis-3 clinical criteria. All eligible patients who underwent 9 routine laboratory tests on the day of admission or the day before were included (Table 1): The primary outcome was in-hospital mortality. Multivariable logistic regression and XGBoost models including age and the 9 labs were trained and evaluated using a 60% training and 40% validation split. Discrimination was assessed with c-statistics, and calibration was evaluated using Brier scores and calibration plots.

**Figure:**
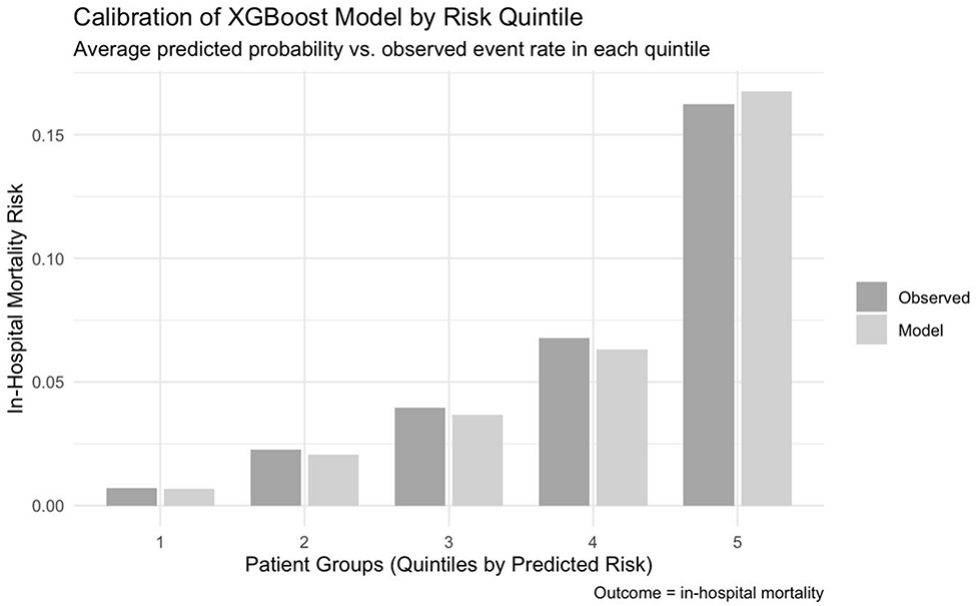
Calibration of XGBoost Model by Risk Quintile Average predicted probability vs. observed event rate in each quintile XGBoost Feature Importance

**Figure:**
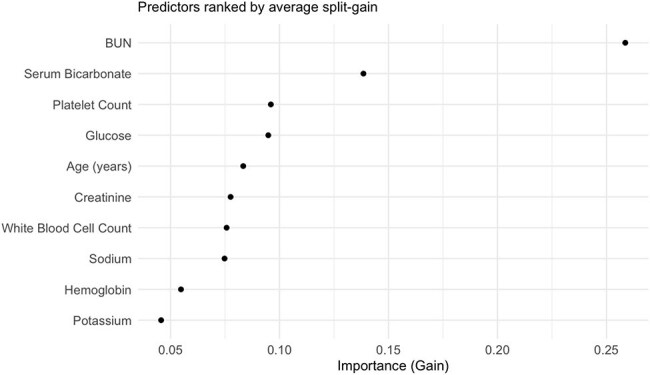
Predictors ranked by average split-gain

**Results:**

Of 724,534 inpatients meeting inclusion criteria, 43,108 (6%) died in-hospital. In the final multivariate model (table 1), low bicarbonate (OR 2.19 [2.19–2.26]) and hypernatremia (OR 2.19 [2.13–2.26]) remained the most significant predictors of mortality. In the final multivariable logistic regression model, the c-statistic was 0.73. The XGBoost model c-statistic was 0.77. Calibration plots for both models demonstrated good agreement between predicted and observed mortality risk.

**Conclusion:**

Among patients with suspected severe infection, routine admission labs and patient age accurately predicted in-hospital mortality and machine learning improved the model. This validation study provides a straightforward, broadly applicable method for severity-of-illness risk adjustment.

**Disclosures:**

Anthony Harris, MD, MPH, UpToDate Wolters Kluwer Health: Infection control section editor

